# Malnutrition and Osteosarcopenia in Elderly Women with Rheumatoid Arthritis: A Dual Clinical Perspective

**DOI:** 10.3390/nu17132186

**Published:** 2025-06-30

**Authors:** Joan M. Nolla, Carmen Moragues, Lidia Valencia-Muntalà, Laia de Daniel-Bisbe, Laura Berbel-Arcobé, Diego Benavent, Paola Vidal-Montal, Antoni Rozadilla, Javier Narváez, Carmen Gómez-Vaquero

**Affiliations:** Department of Rheumatology, IDIBELL-Hospital Universitari de Bellvitge, University of Barcelona, 08907 Barcelona, Spain; cmoragues@bellvitgehospital.cat (C.M.); lvalencia@bellvitgehospital.cat (L.V.-M.); ldedanieli@bellvitgehospital.cat (L.d.D.-B.); lberbel@bellvitgehospital.cat (L.B.-A.); diegobenavent@bellvitgehospital.cat (D.B.); pvidal@bellvitgehospital.cat (P.V.-M.); arozadilla@bellvitgehospital.cat (A.R.); fjnarvaez@bellvitgehospital.cat (J.N.); carmen.gomez@bellvitgehospital.cat (C.G.-V.)

**Keywords:** malnutrition, GLIM criteria, osteosarcopenia, elderly women, rheumatoid arthritis, comorbidity

## Abstract

**Background/Objectives**: Rheumatoid arthritis (RA) is a chronic inflammatory disease frequently accompanied by comorbid conditions that contribute to disability and worsen long-term outcomes. Among these, malnutrition and osteosarcopenia remain under-recognised. This cross-sectional study aimed to assess the prevalence of malnutrition and osteosarcopenia among elderly women with RA and explore the clinical impact of these conditions. **Methods:** Sixty-five women over 65 years with RA were evaluated using Global Leadership Initiative on Malnutrition (GLIM) criteria for malnutrition and EWGSOP2-based assessments for sarcopenia; bone status was measured by dual-energy X-ray absorptiometry (DXA), trabecular bone score (TBS), and three-dimensional DXA (3D-DXA). **Results:** Malnutrition was identified in 49.2% and osteosarcopenia in 52.3% of participants. A significant bidirectional association was observed: malnourished patients had higher rates of osteosarcopenia (65.6% vs. 34.4%; *p* < 0.05), and osteosarcopenic patients were more frequently malnourished (61.8% vs. 39.1%; *p* < 0.05). Both conditions were associated with older age, lower body mass index (BMI), impaired muscle parameters, and reduced bone mineral density. Malnourished and osteosarcopenic patients reported worse fatigue and lower physical quality of life, despite similar inflammatory activity. Significant correlations were found between muscle mass indices and bone quality metrics assessed by 3D-DXA. These findings highlight a substantial burden of malnutrition and osteosarcopenia in elderly women with RA, even with well-controlled disease despite similar inflammatory activity (mean Disease Activity Score 28: 2.8 ± 1.0; 43.1% in remission. **Conclusions:** There is a substantial burden of malnutrition and osteosarcopenia in elderly women with RA that support the integration of systematic nutritional and musculoskeletal screening into routine care. Future studies should evaluate age- and disease-specific mechanisms and assess the benefit of multidisciplinary strategies to prevent frailty and improve long-term outcomes.

## 1. Introduction

Rheumatoid arthritis (RA) is the most frequently diagnosed systemic autoimmune disease worldwide, characterized by persistent and progressive articular and systemic manifestations, leading to a heightened risk of disability and mortality [1]. Recent advances in therapeutic strategies have significantly improved RA outcomes and patient quality of life [2]. These improvements have simultaneously sharpened the focus on comorbidities that may complicate the disease course and impact patient quality of life.

In addition to classical concerns such as cardiovascular disease [3], osteoporosis [4], and infections [5], routine rheumatology practice in the management of RA has increasingly recognized other relevant comorbidities, including anxiety, depression, sexual dysfunction, fatigue, malnutrition, and sarcopenia [6]. Addressing these conditions has become essential to a comprehensive approach to RA management.

Malnutrition is an emerging concern in this context [7]. Patients with RA appear to be particularly vulnerable [8], although the clinical implications of this comorbidity remain incompletely characterized, in part due to considerable variability in reported prevalence, largely influenced by the assessment tools employed.

Historically, the lack of standardized diagnostic criteria hindered reliable identification of malnutrition. In 2018, the Global Leadership Initiative on Malnutrition (GLIM) proposed a set of diagnostic criteria now widely adopted in clinical practice [9]. The GLIM framework includes three phenotypic criteria (weight loss, low body mass index [BMI], and reduced muscle mass) and two etiologic criteria (reduced food intake or assimilation, and the presence of disease burden or inflammation). A diagnosis of malnutrition is established when at least one phenotypic and one etiologic criterion are met.

Sarcopenia, a condition closely linked to malnutrition [10,11] is a progressive and generalized disorder of the skeletal muscle characterized by an accelerated decline in muscle mass and function. It is independently associated with an increased risk of falls, functional impairment, frailty, and mortality [12]. Although no universal definition exists, the revised criteria proposed by the European Working Group on Sarcopenia in Older People in 2019 (EWGSOP2) remain the most commonly applied in clinical and research settings [13]. Using these criteria, recent studies have identified a significant prevalence of sarcopenia among patients with RA, ranging from 15% to 20% [14,15,16].

Osteosarcopenia, first described in 2009 [17], conceptually refers to the coexistence of osteoporosis and sarcopenia. This term emphasizes the functional interdependence of bone and muscle tissues and highlights the compounded adverse consequences when both are concurrently compromised. The synergistic impact of osteoporosis and sarcopenia has been well described [18], reinforcing the need for an integrated approach to diagnosis and management.

A persistent challenge in the field lies in the absence of a standardized definition for osteosarcopenia. Recent literature highlights this heterogeneity, with some studies restricting the diagnosis to individuals meeting criteria for osteoporosis [19], while others adopt broader inclusion criteria encompassing individuals with low bone mineral density [20,21,22]. Similarly, considerations regarding the sarcopenia component vary: some investigations apply the operational criteria proposed by the EWGSOP2 [19,20,21], whereas others employ diverse combinations of deficits in muscle strength, muscle mass, and physical performance to define the condition [22].

At present, no studies have evaluated the prevalence of malnutrition in RA using the GLIM criteria, and data on the frequency of osteosarcopenia in this population are extremely limited [23]. The objective of this study is to address this gap through an exploratory analysis of both conditions in elderly Spanish women with RA, with particular emphasis on the possible associations between these two comorbidities.

## 2. Materials and Methods

### 2.1. Study Design and Participants

**Figure 1:**
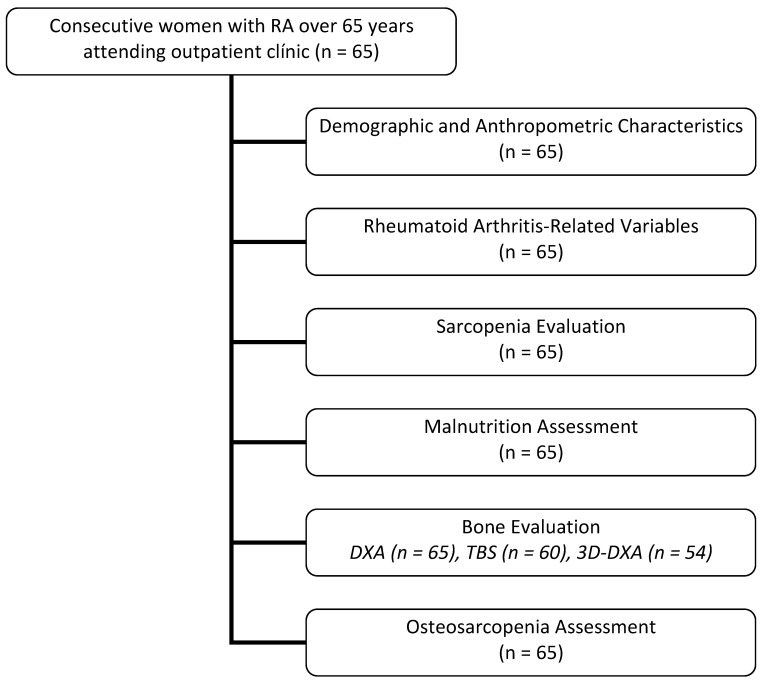
Study flow and data-collection scheme. RA: rheumatoid arthritis. DXA: dual-energy X-ray absorptiometry. TBS: trabecular bone score. 3D-DXA: three-dimensional DXA.

This cross-sectional, observational, exploratory study enrolled female participants aged over 65 years who fulfilled the 2010 ACR classification criteria for RA, as assessed during routine clinical follow-up at a university-affiliated tertiary care rheumatology unit. Patients with conditions that could significantly influence nutritional or functional status—such as malignancies, decompensated cardiac or respiratory disease, or chronic hepatic or renal failure—were excluded. An overview of the study flow and data collection process is provided in Figure 1.

All individuals gave informed written consent prior to enrolment. The study protocol received approval from the institution’s ethics review board (reference: PR057/20).

### 2.2. Data Collection

#### 2.2.1. Demographic and Anthropometric Characteristics

The following baseline characteristics were recorded:

- Age (years).
- Body mass index (BMI), calculated as weight (kg) divided by height squared (m^2^). BMI values were classified as underweight (<18.5 kg/m^2^), normal (18.5–24.9 kg/m^2^), overweight (25–29.9 kg/m^2^), or obese (≥30 kg/m^2^). These BMI categories were defined according to World Health Organization (WHO) criteria.
- Tobacco use. We categorized the patient population into three groups based on tobacco use: never smokers, current smokers, and former smokers.
- Physical activity. We categorized the patient population based on their levels of physical activity into four groups: none, sporadic, regular with low intensity, and regular with high intensity. Low-intensity activity was defined as light efforts such as walking or gardening, while high-intensity activity referred to more vigorous forms such as running, aerobic classes, or recreational sports. Physical activity was considered ‘regular’ when performed at least twice per week.

#### 2.2.2. Rheumatoid Arthritis-Related Variables

- Disease history, including:
  ○ Duration since RA diagnosis (years).
  ○ Current pharmacologic regimen, encompassing glucocorticoids, conventional synthetic disease-modifying antirheumatic drugs (csDMARDs), biologic DMARDs (bDMARDs), and Janus kinase (JAK) inhibitors.
  ○ Positivity of rheumatoid factor (RF), defined as antibody levels exceeding the threshold established by our reference laboratory (≤16 kIU/L).
  ○ Positivity of anti-citrullinated peptide antibodies (ACPA), defined as antibody levels exceeding the threshold established by our reference laboratory (>20 U/mL).
  ○ Laboratory data, based on the most recent available tests:
  ○ Erythrocyte sedimentation rate (ESR).
  ○ C-reactive protein (CRP) concentration.
  ○ Haemoglobin levels.
  ○ Albumin levels.
- Assessment of disease activity was conducted using two validated instruments:
  ○ Disease Activity Score 28 (DAS28) [24], which includes the count of swollen and tender joints (out of 28), ESR, and patient global assessment of disease activity. Interpretation of the score: remission (<2.6), low activity (2.6–3.2), moderate activity (>3.2–5.1), and high disease activity (>5.1).
  ○ Routine Assessment of Patient Index Data 3 (RAPID3) [25], a patient-reported outcome measure combining pain, physical function, and global assessment. Score categories: remission (≤3), low activity (3.1–6), moderate activity (6.1–12), and high activity (>12).
- Functional disability was measured using the Health Assessment Questionnaire (HAQ) [26], with scores ranging from 0 (no disability) to 3 (severe disability).
- Fatigue assessment. The Functional Assessment of Chronic Illness Therapy-Fatigue (FACIT-F) scale [27] was employed to measure fatigue levels. This scale includes items rated on a scale from 0 to 4, yielding a total possible score that ranges from 0 to 52, where lower scores signify greater levels of fatigue.
- Health-related quality of life was evaluated using the 12-Item Short Form Health Survey (SF-12) [28], which measures physical and mental health through eight domains. Two summary scores were derived: a Physical Component Summary and a Mental Component Summary, computed using population-weighted algorithms.

#### 2.2.3. Sarcopenia Evaluation

- Muscle strength was measured using a calibrated Jamar-type digital hand dynamometer (Kern 80K1; KERN & SOHN GmbH, Balingen, Germany). The highest value obtained from two attempts per hand (using the dominant hand) was recorded. Impaired value was defined as grip strength <16 kg.
- Physical performance was assessed via gait speed. Participants were instructed to walk a straight 6-metre path at a comfortable pace, timed with a stopwatch. A gait speed <0.8 m/s was considered impaired
- Skeletal muscle mass was estimated using dual-energy X-ray absorptiometry (DXA) on a Hologic Horizon W device (Hologic Inc., Bedford, MA, USA). Appendicular skeletal muscle mass was indexed to height squared (SMI = ASM/height^2^). An SMI ≤5.67 kg/m^2^ was used as the diagnostic threshold.
- Sarcopenia screening was performed with the SARC-F questionnaire [29], comprising five items: strength, ability to walk, getting up from a chair, climbing stairs, and frequency of falls. Each item is scored from 0 to 2; total scores ≥4 suggest possible sarcopenia and prompt further evaluation.
- Diagnostic classification. Sarcopenia was defined according to EWGSOP-2 [13]. In this way, confirmed sarcopenia was diagnosed when low muscle strength was accompanied by low muscle mass in patients with a SARC-F score ≥ 4.

#### 2.2.4. Malnutrition Assessment

Malnutrition was defined according to the GLIM criteria (6). Reduced muscle mass was assessed using whole-body DXA, as previously described, and was defined by a fat-free mass index (FFMI, kg/m^2^) below 14.6. Low BMI was considered as <20 kg/m^2^, or <22 kg/m^2^ in individuals aged 70 years or older. All participants were considered to meet the etiological criterion of the GLIM framework based on the presence of RA, a chronic inflammatory condition. Data on recent involuntary weight loss were not available.

#### 2.2.5. Bone Evaluation

- Areal Bone Mineral Density (aBMD) Assessment. Bone mineral density (BMD) was measured by DXA using a Horizon Wi densitometer (Hologic Inc., Bedford, MA, USA), with areal BMD (aBMD) values expressed in g/cm^2^. Daily calibration was performed using a lumbar spine phantom, with an in vitro coefficient of variation consistently below 1%. T-scores and Z-scores for the lumbar spine were calculated using reference data from the Spanish Multicentre Research Project on Osteoporosis (MRPO) [30], and those for the proximal femur were based on the NHANES III database [31]. Classification of osteopenia and osteoporosis followed World Health Organization criteria and the official positions of the International Society for Clinical Densitometry [32].
- Trabecular Bone Score (TBS). TBS was derived from lumbar spine DXA scans using TBS iNsight software (version 3.0; Medimaps Group, Plan-les-Ouates, Switzerland). TBS analysis was only performed in patients whose BMI fell within the validated range for TBS interpretation (15–35 kg/m^2^). It was performed in 60 patients. TBS values were interpreted [33] as follows: ≥1.350 (normal microarchitecture), 1.200–1.349 (partially degraded), and ≤1.200 (degraded microarchitecture).
- Three-Dimensional DXA (3D-DXA) Analysis. It was performed using 3D-Shaper-Research software v.2.14 (3D-Shaper Medical, Barcelona, Spain). 3D-DXA analysis was performed retrospectively as we did not have the software installed when the baseline DXA examinations were acquired. Several raw files could not be located, and a few were retrieved but proved irreversibly corrupted. Also, one patient had bilateral total-hip arthroplasties. It was performed in 54 patients. At the total hip, the following parameters were evaluated: cortical surface BMD (sBMD, mg/cm^2^) and trabecular volumetric BMD (vBMD, mg/cm^3^). Classification in normal, low, and very low categories were calculated based on reference data from the Spanish population included in the SEIOMM-3D-DXA project [34].

#### 2.2.6. Definition of Osteosarcopenia

Osteosarcopenia was defined [22] as the coexistence of low bone mineral density—indicated by a T-score <−1.0 at any major skeletal site (lumbar spine, femoral neck, or total hip)—and the presence of at least two of the following criteria: gait speed < 0.8 m/s, grip strength < 16 kg, and SMI ≤ 5.67 kg/m^2^.

### 2.3. Statistical Analysis

Given the exploratory design of the study, aimed at generating preliminary data on the prevalence and potential association between malnutrition and osteosarcopenia in older women with RA, no formal sample size calculation was performed. Continuous variables were tested for normality and expressed as means ± standard deviations or medians with interquartile ranges, as appropriate. Differences between groups for normally distributed variables were assessed using one-way analysis of variance (ANOVA), while the Mann–Whitney U test or Kruskal–Wallis test was applied for non-parametric variables, as appropriate. Categorical variables were compared using the chi-squared test.

To explore associations between key clinical and densitometric parameters, correlation analyses were performed using Pearson’s correlation coefficient.

## 3. Results

The study included 65 elderly women with RA, with a mean age of 72.6 ± 6.3 years and BMI of 27.3 ± 4.8 kg/m^2^. Most were never smokers (87.7%), and physical activity was limited (47.7% inactive). Mean disease duration was 17.9 ± 9.8 years; RF and ACPA were positive in 70.2% and 76.3% of patients, respectively. Glucocorticoids were used by 46.2%, csDMARDs by 87.7%, and bDMARDs by 41.5%. Mean DAS28 was 2.8 ± 1.0, with 43.1% in remission and 1.5% with high disease activity. The mean FFMI was 14.9 ± 2.0 kg/m^2^, and the mean SMI was 5.46 ± 0.80 kg/m^2^. The mean score on the SARC-F questionnaire was 2.95 ± 1.85, with 38.5% of patients (n = 25) scoring ≥4. Sarcopenia was identified in 11 patients (17%).

Bone health parameters included a mean lumbar spine aBMD of 0.905 ± 0.134 g/cm^2^, femoral neck 0.678 ± 0.939 g/cm^2^, total hip 0.862 ± 0.118 g/cm^2^, and a mean TBS of 1.261 ± 0.093. According to WHO criteria, 19 of 65 patients (29.2%) met the diagnostic threshold for osteoporosis, while 37 patients (56.9%) exhibited low BMD. Concurrent osteoporosis and sarcopenia were present in 5 patients (7.7%).

Malnutrition was identified in 49.2% of patients (32/65), while osteosarcopenia was present in 52.3% (34/65). Of the 65 women evaluated, 32 (49%) fulfilled at least one phenotypic GLIM criterion. Seven (10.8%) displayed a low BMI—one aged < 70 years and six aged ≥ 70 years—and all seven simultaneously exhibited a low fat-free mass index (FFMI). An additional 25 women (38.5%) presented with a low FFMI.

A significant bidirectional association was observed between the two conditions. The prevalence of osteosarcopenia was significantly higher among patients with malnutrition compared to those without (65.6% vs. 34.4%; *p* < 0.05). Similarly, malnutrition was more frequent in osteosarcopenic patients than in those without this condition (61.8% vs. 39.1%; *p* < 0.05).

Table 1 presents the clinical, functional, and body composition characteristics of the study population stratified by the presence or absence of malnutrition. Table 2 provides the same variables, stratified according to the presence or absence of osteosarcopenia.

Patients classified as malnourished exhibited a significantly distinct clinical and functional profile compared to those without malnutrition. They were older, had lower BMI, and demonstrated poorer muscle-related parameters, including lower SMI and reduced handgrip strength. Functionally, they reported greater fatigue, as reflected by lower FACIT-F scores, and worse physical quality of life, as indicated by the SF-12 physical component. Moreover, they showed significantly lower BMD at the lumbar spine and total femur, as well as reduced cortical sBMD assessed by 3D-DXA. Notably, the burden persisted in patients with low inflammatory activity: 12 of 28 women in DAS28 remission (42.9%) and 11 of 19 in RAPID3 remission (58.0%) fulfilled ≥1 phenotypic GLIM criterion in addition to the RA-related aetiologic criterion. No significant differences were observed in disease duration, serological status, inflammatory markers, TBS, or trabecular vBMD.

Similarly, patients with osteosarcopenia exhibited a distinct clinical and functional profile compared to those without the condition. They were older, had lower BMI, and showed significantly poorer muscle-related parameters, including reduced SMI and diminished grip strength. In addition, they demonstrated significantly lower BMD at the lumbar spine, femoral neck, and total femur, alongside reduced cortical sBMD and trabecular vBMD. Functionally, these patients reported lower physical quality of life and greater fatigue, despite no significant differences in disease activity, disability scores, inflammatory markers, or TBS.

Correlation analysis demonstrated a strong positive association between FFMI and other nutritional parameters, including BMI (r = 0.884, *p* < 0.01) and SMI (r = 0.742, *p* < 0.01). Notably, FFMI exhibited a modest but statistically significant inverse correlation with serum albumin levels (r = −0.286, *p* < 0.05). No significant associations were observed between FFMI and age, disease duration, serological markers (RF or ACPA), disease activity, disability, health-related quality of life, or fatigue. With respect to bone parameters, FFMI correlated positively with lumbar spine aBMD (r = 0.264, *p* < 0.05) and total hip aBMD (r = 0.335, *p* < 0.01), while no significant correlations were found with femoral neck aBMD, or three-dimensional assessments (cortical sBMD or trabecular vBMD).

Similarly, SMI showed significant positive correlations with BMI (r = 0.725, *p* < 0.001) and haemoglobin levels (r = 0.334, *p* < 0.01). No significant associations were observed between SMI and age, disease duration, serological markers, disease activity, disability, health-related quality of life, or fatigue. In relation to bone parameters, SMI correlated positively with aBMD at all assessed skeletal sites: lumbar spine (r = 0.390, *p* < 0.01), femoral neck (r = 0.402, *p* < 0.01), and total hip (r = 0.514, *p* < 0.001). Additionally, significant associations were observed with three-dimensional bone measures, including cortical sBMD (r = 0.509, *p* < 0.001) and trabecular vBMD (r = 0.305, *p* < 0.05).

## 4. Discussion

The findings of this study emphasize a substantial burden of malnutrition (49.2%) and osteosarcopenia (52.3%) among elderly women with RA, even in the context of low inflammatory activity and preserved functional capacity. This coexistence of subclinical comorbidities reinforces the need to broaden the clinical approach beyond inflammation control, advocating for comprehensive assessments that include nutritional status, muscular involvement, and bone health.

Despite sustained remission being increasingly achievable in RA, non-inflammatory systemic manifestations remain prevalent and impact function and long-term outcomes. Malnutrition, sarcopenia, and bone loss constitute a pathophysiological continuum that may culminate in frailty, particularly in the ageing population. The concomitant decline in muscle and bone, often associated with inadequate intake or absorption, heightens physiologic vulnerability and the risk of adverse outcomes such as falls, hospitalization, and mortality. Early recognition and targeted intervention should be prioritized in the care of this vulnerable subgroup of patients.

To our knowledge, this is the first study to report the prevalence of malnutrition in elderly women with RA using the GLIM criteria. In this cohort, nearly half of the participants met GLIM criteria for malnutrition—a prevalence comparable to that reported in oncologic populations [35] and in patients admitted to acute geriatric wards [36].

The prevalence of osteosarcopenia in this cohort (52.3%) is remarkably high and substantially exceeds the only previously reported estimate in RA. In the study by Tada et al. [23], conducted in a Japanese outpatient population (mean age 66.5 years; 85.7% women), osteosarcopenia was identified in 11% of participants based on the coexistence of sarcopenia, defined according to the AWGS 2014 criteria, and osteoporosis, per Japanese guidelines. The higher prevalence observed in our cohort may be explained by differences in both demographic characteristics and diagnostic approach. Notably, when applying the same strict definition—requiring the coexistence of sarcopenia (per EWGSOP2) and osteoporosis (according to WHO criteria)—the prevalence in our series was 7.7%, closely matching the estimate reported by Tada et al. However, our primary definition of osteosarcopenia, aligned with the conceptual framework first proposed by Huo et al. [22], encompassed low bone mineral density (T-score < −1.0 at any major site) and impairment in two muscle domains (strength, mass, or physical performance). This broader approach increases sensitivity to clinically relevant musculoskeletal deterioration and, in our opinion, may better reflect early or subclinical phenotypes.

In line with this rationale, we deliberately chose a definition that includes both osteopenia and osteoporosis, recognizing that the majority of fragility fractures occur in individuals whose BMD does not meet the formal threshold for osteoporosis [37]. For the muscular component, we moved beyond categorical labels and instead adopted a domain-based framework, requiring at least two impairments among strength, muscle mass, and gait speed. We believe that this model better reflects the progressive nature of musculoskeletal decline and enhances the ability to identify intermediate or transitional phenotypes.

The strong bidirectional association observed between malnutrition and osteosarcopenia in this cohort supports the notion that these conditions are not only co-prevalent but also pathophysiologically intertwined. Patients with malnutrition exhibited significantly higher rates of osteosarcopenia compared to those without (65.6% vs. 34.4%), and similarly, malnutrition was more frequent among individuals with osteosarcopenia (61.8% vs. 39.1%). These findings suggest a mutually reinforcing relationship, whereby nutritional deficits may exacerbate musculoskeletal deterioration, and conversely, declining bone and muscle integrity may contribute to a catabolic state that accelerates undernutrition.

Several mechanisms [38,39] may explain this interaction. Chronic systemic inflammation—present even in clinically controlled RA—can promote protein-energy catabolism, suppress appetite, and impair nutrient absorption. Pro-inflammatory cytokines such as TNF and IL-6 are known to inhibit both myogenesis and osteoblastogenesis while enhancing muscle proteolysis and bone resorption.

Beyond prevalence estimates, this study contributes novel insights into the relationship between nutritional status, muscle parameters, and bone health in elderly women with RA. Specifically, we observed significant positive correlations between FFMI and aBMD at the lumbar spine and total hip, as well as between SMI and aBMD across all measured sites. These findings highlight the close anatomical and metabolic interdependence between muscle and bone tissues and support the relevance of muscle quantity—often compromised in malnutrition—as a determinant of skeletal integrity.

Notably, this is the first study in RA to explore the association between muscle parameters and advanced bone quality metrics, including TBS and 3D-DXA. SMI correlated significantly with both cortical sBMD and trabecular vBMD, underscoring the muscle–bone interaction. A key finding is the significantly lower cortical sBMD in malnourished patients compared to those without malnutrition (138.7 ± 23.6 vs. 155.5 ± 21.9 mg/cm^2^, *p* < 0.01), with no significant difference in trabecular vBMD. This suggests that malnutrition predominantly affects the cortical compartment at the hip. Given that cortical bone contributes disproportionately to hip strength and is a primary determinant of resistance to bending and torsional forces [40], this localized deterioration may underlie the increased susceptibility to hip fractures observed in frail populations.

Both malnutrition and osteosarcopenia were associated with clinically relevant impairments in this cohort. Malnourished patients exhibited lower BMI and reduced SMI, alongside greater fatigue and poorer physical quality of life, as reflected in FACIT-F and SF-12 scores. Likewise, individuals with osteosarcopenia showed a more compromised musculoskeletal profile, with deficits in muscle mass, strength, and bone quality (3D-DXA parameters) paralleled by worse patient-reported outcomes. Notably, these associations emerged despite comparable levels of inflammatory activity and disability, suggesting that nutritional and structural deterioration contribute independently to functional decline in RA.

This study has several limitations. Its cross-sectional design precludes causal inference, and no formal sample size calculation was performed due to the exploratory nature of the study and the lack of prior data on malnutrition and osteosarcopenia prevalence in this specific population. The single-centre setting and relatively small cohort size may limit generalizability, and no data were collected on dietary intake [41], which could have enriched the interpretation of the findings. Moreover, as age-related changes include loss of lean mass and nutritional decline, we acknowledge that our results might reflect ageing. Even so, the 49% malnutrition prevalence in our cohort is two- to four-fold higher than that reported for community-dwelling women of comparable age [42,43,44], suggesting an additional disease-related burden. Regarding osteosarcopenia, population-based evidence is still limited; therefore, we cannot rule out that our findings reflect age-related changes rather than RA itself.

Among its strengths, this study is the first to assess malnutrition in elderly women with RA using GLIM criteria and to characterize osteosarcopenia through a combined structural and functional approach, incorporating DXA, TBS, and 3D-DXA-derived parameters alongside EWGSOP2-based muscle assessment. The use of validated patient-reported outcomes adds clinical relevance, while the focus on a homogeneous, well-defined population enhances internal consistency and reduces potential confounding.

Future research should aim to disentangle the respective contributions of ageing and RA to the development of malnutrition and osteosarcopenia. Prospective cohorts of autoantibody-positive individuals without clinical arthritis (pre-RA) could also illuminate early nutritional derangements. Comparative studies in younger, premenopausal women with RA could help clarify the role of age-related versus disease-related mechanisms. Longitudinal studies are also needed to understand the temporal evolution and clinical consequences of these conditions. Finally, our findings underscore the importance of multidisciplinary strategies for patients identified as high-risk, including early referral to dietitians, implementation of tailored physiotherapy programs, and consideration of pharmacological bone protection where appropriate.

## 5. Conclusions

This study demonstrates the substantial burden and clinical significance of malnutrition and osteosarcopenia in elderly women with rheumatoid arthritis. These conditions were not only common and closely interrelated but also independently associated with reduced muscle mass and strength, lower bone quality, increased fatigue, and poorer physical quality of life—even in the context of low inflammatory activity. The results support the integration of nutritional screening and comprehensive musculoskeletal assessment into routine care for older patients with RA. Early recognition may allow timely interventions to prevent or attenuate frailty and its associated outcomes. Further research is needed to validate these findings in more diverse populations and to explore the benefits of multidisciplinary strategies in this vulnerable group.

## Figures and Tables

**Table 1:** Patient characteristics in accordance with the presence of malnutrition.

	All Patients (n: 65)	Without Malnutrition (n: 33)	With Malnutrition (n: 32)	*p*
**Age (years)**	72.6 ± 6.3	72.7 ± 5.5	72.5 ± 7.0	ns
**BMI (kg/m^2^)**	27.3 ± 4.8	30.8 ± 3.7	23.8 ± 2.8	<0.001
** Underweight (n, %)**	0	0	0	
** Normal range (n, %)**	23 (35.4%)	1 (3.0%)	22 (68.8%)	
** Overweight (n, %)**	24 (36.9%)	15 (45.5%)	9 (28.1%)	
** Obese (n, %)**	18 (27.7%)	17 (51.5%)	1 (3.1%)	<0.001
**Tobacco use**				
** Never**	57 (87.7%)	29 (87.9%)	28 (87.5%)	
** Ever**	8 (12.3%)	4 (2.1%)	4 (12.5%)	ns
**Physical activity**				
** No**	31 (47.7%)	14 (42.4%)	17 (53.1%)	
** Sporadic**	13 (20.0%)	8 (24.3%)	5 (15.6%)	
** Regular with low intensity**	21 (32.3%)	11 (33.3%)	10 (31.3%)	ns
**Disease duration (years)**	17.9 ± 9.8	18.3 ± 9.9	17.4 ± 9.7	<0.05
**Current medication**				
** Glucocorticoids (n, %)**	30 (46.2%)	18 (54.5%)	12 (37.5%)	ns
** cDMARDs (n, %)**	57 (87.7%)	30 (90.9%)	27 (84.4%)	ns
** bDMARDs (n, %)**	27 (41.5%)	11 (33.3%)	16 (50.0%)	ns
** Jak inhibitors (n, %)**	1 (1.5%)	0	1 (3.1%)	ns
**RF seropositivity (n, %)**	40 (70.2%)	22 (75.9%)	18 (64.3%)	ns
** RF titer**	150.3 ± 271.4	201.4 ± 383.6	106.2 ± 98.7	ns
**ACPA seropositivity (n, %)**	45 (76.3%)	26 (83.9%)	19 (67.9%)	ns
** ACPA titer**	249.5 ± 379.4	181.8 ± 208.5	335.1 ± 516.2	ns
**ESR (mm/h)**	22.7 ± 16.6	22.6 ± 15.9	22.8 ± 17.5	ns
**CRP (mg/dL)**	4.70 ± 7.0	5.2 ± 6.5	4.2 ± 7.6	ns
**Haemoglobin (g/dL)**	13.5 ± 1.0	13.5 ± 1.1	13.4 ± 0.9	ns
**Albumin (g/L)**	43.9 ± 3.8	43.2 ± 4.0	44.6 ± 3.4	ns
**DAS28**	2.8 ± 1.0	2.8 ± 1.0	2.8 ± 1.0	ns
** Remission (n, %)**	28 (43.1%)	16 (48.5%)	12 (37.5%)	
** Low disease activity (n, %)**	20 (30.8%)	8 (24.2%)	12 (37.5%)	
** Moderate disease activity (n, %)**	16 (24.6%)	8 (24.2%)	8 (25.0%)	
** High disease activity (n, %)**	1 (1.5%)	1 (3.0%)	0 (0%)	ns
**RAPID3**	9.7 ± 7.4	11.2 ± 7.5	8.2 ± 7.1	ns
** Remission (n, %)**	19 (30.2%)	8 (25.0%)	11 (35.5%)	
** Low disease activity (n, %)**	3 (4.8%)	0 (0%)	3 (9.7)	
** Moderate disease activity (n, %)**	19 (30.2%)	10 (31.3%)	9 (29.0%)	
** High disease activity (n, %)**	22(34.9%)	14 (43.8%)	8 (25.8%)	ns
**HAQ**	0.15 ± 0.34	0.23 ± 0.47	0.07 ± 0.09	ns
**FACIT-F**	35.4 ± 9.9	33.6 ± 10.3	37.2 ± 9.2	ns
**SF-12**				
** Mental health**	44.5 ± 11.4	42.9 ± 11.5	46.1 ± 11.3	ns
** Physical health**	37.5 ± 9.2	36.7 ± 9.7	38.3 ± 8.8	ns
**Grip strength < 16 g (n, %)**	39/65 (60.0%)	21 (63.6%)	18 (56.3%)	ns
**Gait speed < 0.8 m/s (n, %)**	18 (27.7%)	11 (33.3%)	7 (21.9%)	ns
**SMI**	5.46 ± 0.80	5.96 ± 0.56	4.94 ± 0.66	<0.001
** SMI ≤ 5.67 Kg/m^2^ (n, %)**	40 (61.5%)	10 (30.3%)	30 (93.8%)	<0.001
**FFMI, Kg/m^2^**	14.9 ± 2.0	16.4 ± 1.3	13.3 ± 1.1	<0.001
**Lumbar aBMD (g/cm^2^)**	0.905 ± 0.134	0.949 ± 0.125	0.856 ± 0.131	<0.01
**Femoral neck aBMD (g/cm^2^)**	0.678 ± 0.939	0.700 ± 0.088	0.658 ± 0.097	ns
**Total femur aBMD (g/cm^2^)**	0.862 ± 0.118	0.914 ± 0.101	0.811 ± 0.113	<0.001
**Trabecular Bone Score**	1.261 ± 0.093	1.262 ± 0.095	1.261 ± 0.093	ns
** Normal**	21 (35%)	10 (26%)	11 (35%)	
** Partially degraded**	14 (23%)	16 (41%)	8 (26%)	
** Totally degraded**	25 (42%)	13 (33%)	12 (39%)	ns
**Cortical sBMD, mg/cm^2^**	146.77 ± 24.10	155.46 ± 21.92	138.70 ± 23.57	<0.01
** Normal cortical sBMD**	27 (50%)	19 (73%)	8 (28%)	
** Low cortical sBMD**	23 (43%)	6 (23%)	17 (61%)	
** Very low cortical sBMD**	4 (7%)	1 (4%)	3 (11%)	<0.01
**Trabecular vBMD, mg/cm^3^**	157.14 ± 31.87	165.67 ± 29.47	149.22 ± 32.47	ns
** Normal trabecular vBMD**	24 (44%)	16 (61%)	8 (29%)	
** Low trabecular vBMD**	27 (50%)	9 (35%)	18 (64%)	
** Very low trabecular vBMD**	3 (6%)	1(4%)	2(7%)	<0.01

BMI, body mass index; RF, rheumatoid factor; ACPA, anti-citrullinated peptides antibodies; ESR, erythrocyte sedimentation rate; CRP, C-reactive protein; DAS28, Disease Activity Score 28; RAPID3, Routine Assessment of Patient Index Data 3; HAQ, Health Assessment Questionnaire; cDMARDs, conventional disease-modifying antirheumatic drugs; bDMARDs; biological disease-modifying antirheumatic drugs; FACIT-F: Functional Assessment of Chronic Illness Therapy-Fatigue scale. SF-12: the 12-Item Short Form Health Survey; SMI, Skeletal Mass Index. FFMI: Fat-free mass index. BMD: bone mineral density; aBMD, areal BMD; sBMD, surface BMD; vBMD, volumetric BMD. ns, not significant.

**Table 2:** Patient characteristics in accordance with the presence of osteosarcopenia.

	All Patients (n: 65)	Without Osteosarcopenia (n: 31)	With Osteosarcopenia (n: 34)	*p*
**Age (years)**	72.6 ± 6.3	70.2 ± 4.6	74.8 ± 6.8	<0.01
**BMI (kg/m^2^)**	27.3 ± 4.8	28.9 ± 5.2	25.9 ± 4.0	<0.05
** Underweight (n, %)**	0	0	0	
** Normal range (n, %)**	23 (35.4%)	8 (25.8%)	15 (44.1%)	
** Overweight (n, %)**	24 (36.9%)	10 (32.2%)	14 (41.1%)	
** Obese (n, %)**	18 (27.7%)	13(42.0%)	5 (14.8%)	<0.05
**Tobacco use**				
** Never**	57 (87.7%)	27 (87.1%)	30 (88.2%)	
** Ever**	8 (12.3%)	4 (12.9%)	4 (11.8%)	ns
**Physical activity**				
** No**	31 (47.7%)	10 (32.2%)	21 (61.8%)	
** Sporadic**	13 (20.0%)	7 (22.6%)	6 (17.6%)	
** Regular with low intensity**	21 (32.3%)	14 (45.2%)	7 (20.6%)	<0.05
**Disease duration (years)**	17.9 ± 9.8	16.2 ± 10.2	19.3 ± 9.2	ns
**Current medication**				
** Glucocorticoids (n, %)**	30 (46.2%)	11 (35.5%)	19 (55.9%)	ns
** cDMARDs (n, %)**	57 (87.7%)	30 (96.8%)	27 (79.4%)	<0.05
** bDMARDs (n, %)**	27 (41.5%)	10 (32.3%)	17 (50.0%)	ns
** Jak inhibitors (n, %)**	1 (1.5%)	0	1 (3.0%)	ns
**RF seropositivity (n, %)**	40 (70.2%)	19 (65.5%)	21 (75.0%)	ns
** RF titer**	150.3 ± 271.4	175.0 ± 340.1	129.0 ± 201.2	ns
**ACPA seropositivity (n, %)**	45 (76.3%)	23 (74.2%)	22 (64.7%)	ns
** ACPA titer**	249.5 ± 379.4	135.3 ± 132.7	369.1 ± 504.4	<0.05
**ESR (mm/h)**	22.7 ± 16.6	22.2 ± 16.2	23.1 ± 17.1	ns
**CRP (mg/dL)**	4.70 ± 7.0	3.5 ± 3.1	5.7 ± 9.2	ns
**Haemoglobin (g/dL)**	13.5 ± 1.0	13.9 ± 0.9	13.1 ± 0.9	<0.01
**Albumin (g/L)**	43.9 ± 3.8	43.8 ± 4.3	43.9 ± 3.3	ns
**DAS28**	2.8 ± 1.0	2.6 ± 0.9	3.0 ± 1.0	ns
** Remission (n, %)**	28 (43.1%)	17 (54.8%)	11 (32.3%)	
** Low disease activity (n, %)**	20 (30.8%)	8 (25.8%)	12 (35.3%)	
** Moderate disease activity (n, %)**	16 (24.6%)	6 (19.4%)	10 (29.4%)	
** High disease activity (n, %)**	1 (1.5%)	0	1 (3.0%)	ns
**RAPID3**	9.7 ± 7.4	8.6 ± 7.5	10.7 ± 7.3	ns
** Remission (n, %)**	19 (30.2%)	11 (37.9%)	8 (23.5%)	
** Low disease activity (n, %)**	3 (4.8%)	1 (3.4%)	2 (5.9%)	
** Moderate disease activity (n, %)**	19 (30.2%)	8 (27.6%)	11 (32.3%)	
** High disease activity (n, %)**	22(34.9%)	9 (31.1%)	13 (38.3%)	ns
**HAQ**	0.15 ± 0.34	0.08 ± 0.10	0.23 ± 0.46	ns
**FACIT-F**	35.4 ± 9.9	36.6 ± 10.8	34.3 ± 9.0	ns
**SF-12**				
** Mental health**	44.5 ± 11,4	46.4 ± 10.9	42.7 ± 11.7	ns
** Physical health**	37.5 ± 9.2	38.0 ± 9.9	37.1 ± 8.7	ns
**Grip strength < 16 g (n, %)**	39 (60.0%)	8 (25.8%)	31 (91.2%)	<0.001
**Gait speed < 0.8 m/s (n, %)**	18 (27.7%)	2 (6.5%)	16 (47.1%)	<0.001
**SMI**	5.46 ± 0.80	5.71 ± 0.61	5.21 ± 0.86	<0.05
** SMI ≤ 5.67 Kg/m^2^ (n, %)**	40 (61.5%)	14 (45.2%)	26 (76.5%)	<0.01
**FFMI, Kg/m^2^**				
**Lumbar spine aBMD (g/cm^2^)**	0.905 ± 0.134	0.944 ± 0.138	0.868 ± 0.122	<0.05
**Femoral neck aBMD (g/cm^2^)**	0.678 ± 0.939	0.716 ± 0.085	0.645 ± 0.089	<0.01
**Total hip aBMD (g/cm^2^)**	0.862 ± 0.118	0.917 ± 0.104	0.813 ± 0.108	<0.001
**Trabecular Bone Score**	1.261 ± 0.093	1.268 ± 0.099	1.255 ± 0.088	ns
** Normal**	21 (35%)	10 (37%)	11 (33%)	
** Partially degraded**	14 (23%)	5 (19%)	9 (27%)	
** Totally degraded**	25 (42%)	12 (44%)	13 (40%)	ns
**Cortical sBMD, mg/cm^2^**	146.77 ± 24.10	156.92 ± 21.68	137.34 ± 22.67	<0.01
** Normal cortical sBMD**	27 (50%)	17 (65%)	10 (36%)	
** Low cortical sBMD**	23 (43%)	9 (35%)	14 (50%)	
** Very low cortical sBMD**	4 (7%)	0	4 (14%)	<0.05
**Trabecular vBMD, mg/cm^3^**	157.14 ± 31.87	167.83 ± 31.0	147.22 ± 29.86	<0.05
** Normal trabecular vBMD**	24 (44%)	17 (65%)	7 (25%)	
** Low trabecular vBMD**	27 (50%)	9 (35%)	18 (64%)	
** Very low trabecular vBMD**	3 (6%)	0	3 (11%)	<0.01

BMI, body mass index; RF, rheumatoid factor; ACPA, anti-citrullinated peptides antibodies; ESR, erythrocyte sedimentation rate; CRP, C-reactive protein; DAS28, Disease Activity Score 28; RAPID3, Routine Assessment of Patient Index Data 3; HAQ, Health Assessment Questionnaire; cDMARDs, conventional disease-modifying antirheumatic drugs; bDMARDs; biological disease-modifying antirheumatic drugs; FACIT-F: Functional Assessment of Chronic Illness Therapy-Fatigue scale. SF-12: the 12-Item Short Form Health Survey; SMI, Skeletal Mass Index. FFMI: Fat-free mass index. BMD: bone mineral density; aBMD, areal BMD; TBS: trabecular bone score; sBMD, surface BMD; vBMD, volumetric BMD. ns, not significant.

## Data Availability

The data presented in this study are available on request from the corresponding author. The data are not publicly available due to privacy restrictions.
